# Prevalence of Potentially Traumatic Events, Other Life Events and Subsequent Reactions Indicative for Posttraumatic Stress Disorder in the Netherlands: A General Population Study Based on the Trauma Screening Questionnaire

**DOI:** 10.3390/ijerph17051725

**Published:** 2020-03-06

**Authors:** Jeroen Knipscheer, Marieke Sleijpen, Laurence Frank, Ron de Graaf, Rolf Kleber, Margreet ten Have, Michel Dückers

**Affiliations:** 1 ARQ National Psychotrauma Centre, 1112 XE Diemen, The Netherlands; m.sleijpen@centrum45.nl (M.S.); l.e.frank@uu.nl (L.F.); r.kleber@uu.nl (R.K.); m.duckers@nivel.nl (M.D.); 2 Department of Methodology and Statistics, Utrecht University, 3584 CS Utrecht, the Netherlands; 3 Netherlands Institute of Mental Health and Addiction, 3521 VS Utrecht, The Netherlands; rgraaf@trimbos.nl (R.d.G.); mhave@trimbos.nl (M.t.H.); 4 Nivel–Netherlands Institute for Health Services Research, 3513 CR Utrecht, The Netherlands

**Keywords:** epidemiology, posttraumatic stress disorder, prevalence, gender, ethnicity

## Abstract

The 12-month and lifetime prevalence of posttraumatic stress disorder (PTSD) in different country populations has been assessed while using clinical interviews. Because this methodology is relatively time-consuming and resource-intensive, disaster health researchers adopted instruments, like the Trauma Screening Questionnaire (TSQ). This study (1) used the TSQ to estimate the lifetime prevalence of potentially traumatic events and other life events (PTE/OLEs) and the one-week prevalence of subsequent reactions indicative for PTSD (based on DSM-IV PTSD criteria) in The Netherlands and (2) investigated risk and protective factors for the development of PTSD to overcome the lack of baseline comparison data on general populations and subgroups. The data were derived from the Netherlands Mental Health Survey and Incidence Study-2 (NEMESIS-2), a representative study in the Dutch general population aged 18 to 64 years (N = 6646), using face-to-face interviews. Logistic regression modeling was used to assess PTSD correlates. The lifetime PTE/OLE prevalence was 71.1%. Among exposed subjects, one-week PTSD prevalence was estimated at 2.0%. The correlates of PTSD were female gender, Moroccan, or Turkish ethnicity, and exposure to sexual abuse and exposure time less than four years ago. The results are discussed in relation to earlier 12-month and lifetime general population prevalence of PTSD in the Netherlands and other countries, and TSQ-based disaster studies. General population replications can provide additional TSQ baseline data, and shed light on exposure and PTSD prevalence assessed with different instruments.

## 1. Introduction

Most people report at least one potentially traumatic event (PTE) during their lifetime. Such an event has to refer to being exposed to actual or threatened death, severe injury, or sexual violence, according to the DSM-5 Criterion A. Examples are traffic accident, rape, armed robbery, or traumatic loss of loved one. However, people may also be seriously affected by other life events (OLEs), such as divorce or being fired. Benjet and colleagues reported that over 70% of the respondents were exposed to at least one PTE in their lifetime based on an analysis of 29 PTE types in 24 countries; 30.5% reported exposure to four or more PTEs. Half of all exposures were linked to witnessing death or severe injury, the unexpected death of a loved one, being mugged, being in a life-threatening traffic accident, and experiencing a life-threatening illness or injury. Being married was the most consistent protective factor. Exposure to interpersonal violence had the strongest associations with subsequent traumatic events [1]. In another study a similar average lifetime PTE exposure rate in 16 countries was reported: 67% [2].

However, only a minority develops posttraumatic stress disorder (PTSD) [3,4]. The lifetime prevalence of PTSD varies widely across epidemiologic studies, with rates that range from less than 1% (e.g., Nigeria [5] or Switzerland [6]) up to approximately 5–9% (in the United States [7], the Netherlands [8], and Norway [9]), and as high as 37% in post conflict countries (e.g., Liberia, Algeria, and Cambodia [10]). Variations in prevalence may be largely attributed to deviating risks of exposure to potentially traumatic events and the type of event (e.g., physical assault versus war exposure), to differences in the levels of wealth and vulnerability expressed in a variety of socioeconomic country characteristics [2,11,12], but also to differences in study methodology, i.e., differences in PTE definition, time frame reference (lifetime versus six-month or one-week), assessment strategies for PTSD, and population sampling [8,13]. Even in comparison studies that are based on similar research methods and instruments, substantial differences have been found in the prevalence of PTSD and other mental health problems [2,14,15].

A number of risk and resilience factors influence individual differences in reactions. Multivariate studies indicate that there is no single dominant predictor of PTSD outcome. Most of the predictor variables exert small to moderate effects, and it is the combination or additive total of risk and resilience factors that informs PTSD outcomes. Demographic groups that have consistently been associated with a higher risk of psychopathology (including PTSD) following traumatic exposure are females, children, and older adults, and members of ethnic minority groups [1,6,16,17,18]. However, the impact of ethnicity is complicated by its confounding overlap with socioeconomic status and other risk and resilience factors. Studies that fail to control for such confounding factors generally report significant ethnic effects, while studies using multivariate modeling to control for socioeconomic indicators report often null effects for ethnicity [16].

A methodological issue of scientific as well as practical concern is that population studies that are based on clinical diagnostic interviews are problematic in the aftermath of a disaster or war. Disaster settings are characterized by devastation and disruption, chaos and human loss, and they are accompanied by substantial mental and physical health problems [16,19,20,21]. The circumstances for assessments are suboptimal, especially when populations are relocated to temporary housing or camps, despite the recommended need to be responsive to these problems [22,23,24]. Moreover, clinical diagnostic interviews—a common approach in population studies—are relatively time-consuming and resource-intensive. Screening instruments for PTSD symptoms, like the Trauma Screening Questionnaire (TSQ), are considerably shorter and have been used in recent years to assess trauma-related psychopathology in disaster and related settings [25,26,27,28]. However, baseline general population data, preferably on vulnerable or risk groups, such as ethnic minorities and people with lower socioeconomic status, are scarce but invaluable, in order to interpret the prevalence of symptoms and disorders under particular conditions. Moreover, it is relevant to gain a proper understanding of the prevalence of symptoms in relation to these other life events, given the likely psychosocial impact of other life events (OLEs, such as divorce and being fired), and given their relevance as additional risk factors/stressor in combination with exposure to PTEs (other authors have not even distinguished between the two types of events; e.g., Kessler and colleagues [7]).

The objectives of this study are to estimate in the Dutch population: (1) the lifetime prevalence of exposure to potentially traumatic and other life events (PTEs/OLEs), categorized by type and gender, (2) the current (one-week) prevalence of subsequent reactions indicative for PTSD categorized by type of event, and (3) the associations of risk and protective factors for the development of reactions that are indicative for PTSD (i.e., gender, age, educational level, employment status, ethnicity, type of PTE/OLE, and time passed since exposure).

## 2. Materials and Methods

This study analyzed data from the first wave of the Netherlands Mental Health Survey and Incidence Study-2 (NEMESIS-2) among a representative sample of Dutch adults, performed between November 2007 and July 2009. A comprehensive description of the study aims, sample procedure, diagnostic instruments, and quality control procedures have been documented elsewhere [29].

### 2.1. Setting and Recruitment

In NEMESIS-2 a multistage, stratified random sampling procedure was applied. First, a random sample of municipalities was drawn. Second, a random sample of the addresses of private households from postal registers in these municipalities was drawn, each address with the same probability of selection. Third, a random individual aged 18–64 years and sufficiently fluent in the Dutch language was selected to participate in a face-to-face interview based on the most recent birthday at first contact within the household. The addresses of institutions were excluded; thus institutionalized individuals (i.e., those living in hospices, prisons) were excluded. However, those temporarily living in institutions could be interviewed later during the fieldwork if they returned home. The Minister of Health, Welfare, and Sport sent selected households a letter, in which he explained and recommended the study. A brochure was accompanied explaining its goals in more detail and referring to a website for respondents. Shortly after sending out this letter, households were contacted by telephone or visited in person if no phone number was available.

The Medical Ethics Review Committee for Institutions on Mental Health Care (METIGG) approved the study proposal, field procedures, and information for respondents.

### 2.2. Sample

In the first wave 6646 persons aged 18–64 were included (response rate 65.1%). The sample was reasonably nationally representative, although younger subjects were somewhat underrepresented. A weighting factor was constructed to correct for different response rates in different population groups to be able to generalize the results to the general population.

The estimates of lifetime prevalence of PTE/OLE type and PTE/OLE exposure are based on N = 6457 respondents of the entire sample of 6646 participants, because 189 did not provide valid information (140 respondents received the shortened NEMESIS-2 face-to-face interview without the PTSD section and 49 respondents who took part in the entire interview, including the PTSD section did not provide complete answers to the PTSD questionnaire).

The estimates of current (one-week) prevalence of PTSD are based on N = 4639 respondents. The number 4639 corresponds to the number respondents who provided valid answers and who reported at least one PTE/OLE. Concerning risk and protective factors: sociodemographic (e.g., gender, age, educational level, employment situation, and ethnicity) and conditional (type of event and time passed since exposure) correlates of PTSD were based on N = 4545 respondents, because of missing values on the sociodemographic variables.

### 2.3. Instruments

For this paper the following data were used:

1. PTE/OLEs: The participants were first asked which event most deeply intervened during his or her entire life. Ten events were prescribed: six PTEs in line with the A1 criterion of DSM-IV (Severe traffic accident; Violent robbery; Physical abuse; Sexual abuse; (natural) Disaster; War-related situation) and four OLEs (Loss of own child; Loss of partner; Divorce; Being fired). Item 11 concerned ‘other events’, in which case the respondent was asked to specify. In addition, it was inquired at what age this event took place and how much impact it had (varying from slightly (1) to very (4)). The answers in the box “other events” were categorized by the first three authors, together with assistants: all of the answers were assessed though some answers did not fit in any category (see Table 1).
2. Subsequent reactions indicative for PTSD: PTSD symptoms were measured in response to the most important event, as mentioned by the respondent, with the TSQ. The TSQ is a validated 10-item symptom screener that was designed for use for survivors of all types of traumatic stress [30,31]. The TSQ is based on items from the PTSD Symptom Scale—Self Report (PSS-SR) [32]. It has five re-experiencing items and five arousal items identified by criterion B and D of the DSM-IV PTSD diagnosis [33]. Re-experiencing is tapped with the following items: ‘upsetting thoughts or memories about the event that have come into your mind against your will’, ‘upsetting dreams about the event’, ‘acting or feeling as though the event is/was happening again’, ‘feeling upset by reminders of the event’, and ‘bodily reactions (such as fast heartbeat, stomach churning, sweatiness, dizziness) when reminded of the event’. The arousal items are: ‘difficulty falling or staying asleep’, ‘irritability or outbursts of anger’, ‘difficulty concentrating’, ‘heightened awareness of potential dangers to yourself and others’, and ‘being jumpy or being startled at something unexpected’. The participants were asked whether or not they had experienced each symptom at least twice in the past week. The one-week measure indicated the level of PTSD reactions at the time the respondent completed the survey (regardless of when the event occurred). The Dutch version of the TSQ was reported to be a useful instrument for identifying cases of PTSD: indices of psychometric quality (specificity, sensitivity, reliability, and validity) were satisfactory [34]. Brewin et al. considered the screen “positive” when at least six items were endorsed [30]. In accordance, in the current study, a cut-off score of six was used as indication of being at risk for PTSD. Evidently, the screening instruments are not intended for full diagnosis, and we therefore only might find a “probable PTSD”. However, throughout this manuscript, we refer to this indication by the term PTSD.
3. Sociodemographic characteristics: Gender, age, educational attainment, country of birth, living situation, and employment situation (for specific categories, see Table 3).
4. Event related correlates of PTSD: The correlates were ‘type of event (each PTE/OLE)’ and ‘time passed since exposure’ (measured by the difference between the current age of the respondent and the age at onset of the PTE/OLE).

### 2.4. Statistical Analyses

The cross-tabulations were used to explore the prevalence estimates of PTE/OLEs and indications for PTSD; logistic regression analysis was used to test the association between PTSD and risk and protective factors. An additional logistic regression analysis took the time passed since the PTE/OLE into account. For subjects with PTSD, the median time (Mdn) passed since the PTE/OLE was two years (interquartile range IQR = 9 years), while for subjects not at risk, the time passed since the PTE/OLE was longer with Mdn = 9 (IQR = 15 years). Time passed since the PTE/OLE occurred was categorized in three levels based on tertiles: <4 years ago, four to nine years ago and more than nine years ago. All of the statistical analyses were performed while using the Complex Samples module of IBM SPSS Statistics version 20 to take strata and sampling weights into account.

## 3. Results

### 3.1. Lifetime Prevalence of Exposure to Potentially Traumatic Events and Other Life Events

The lifetime prevalence of exposure to potentially traumatic and other life events was 71.1% (SE = 0.9%). The estimated lifetime prevalence of exposure to potentially traumatic events according to DSM-IV criterion A was 43.8%. Women had significant higher odds of experiencing traumatic events than men (OR = 1.32). Table 1 displays prevalence of PTE/OLEs for men and women, and the gender differences for each event type. The most frequently reported event was “loss of loved one” (other than child or partner) (26.4%); followed by “divorce” (8.6%) and “illness of loved one” (7.1%). Women had almost five times higher odds of reporting “sexual abuse” than men, and about twice higher odds of reporting “not able to get children” as the most traumatic event. In addition, women had 1.5 times higher odds of reporting “losing their partner” and of “illness of loved one”. When compared to women, men had two times higher odds of reporting “serious socio-economic problems” as the most traumatic event, “being fired”, being involved in a “severe traffic accident”, or reporting an event in the category “other”.

### 3.2. Prevalence Estimates of Subsequent Reactions Indicative for PTSD and the Association with Type of Event

From all of the exposed respondents (N = 4667), 2.0% (95% CI = 1.6%–2.7%) reported scores on the TSQ that was indicative for PTSD. Table 2 presents the association between type of PTE/OLE and probable PTSD symptomatology. The odds of being a PTSD case is significantly higher for respondents reporting “serious relational problems” (OR = 4.75; 95% CI = 1.83–12.34) or “sexual abuse” as the most traumatic or other life event (OR = 3.52; 95% CI = 1.64–7.52) and significantly lower for respondents reporting “divorce” or “loss of loved one (other than child or partner)”.

### 3.3. The Association between Sociodemographic Characteristics and Type of Event with PTSD Symptoms

Table 3 shows the prevalence of sociodemographic characteristics and PTE/OLEs, and associations between sociodemographic characteristics, PTE/OLEs, and PTSD, adjusted for time. The results in Table 3 show that the following characteristics are significantly associated with an increased odds of developing PTSD: female gender, age category 45–54 as compared to 55–64, Moroccan or Turkish ethnicity and sexual abuse. The respondents in the lower education categories (primary and lower secondary) show higher odds of developing PTSD when compared to respondents in the higher professional education category, but the differences in odds are not statistically significant. However, the respondents in the higher secondary category show significantly lower odds of developing PTSD when compared to the respondents in the higher professional education category.

The adjusted odds ratio shows that when the event occurred less than four years ago, the odds for having PTSD increases almost five times, as compared to the category, where the event occurred more than nine years ago.

## 4. Discussions

This study provides information regarding the lifetime prevalence of exposure to potentially traumatic and other life events, the one-week prevalence of PTSD, and the relevance of specific risk and protective factors in the Dutch general population. In sum, the lifetime prevalence of exposure to PTEs was 43.8% and the prevalence including OLEs was 71.1%. The most frequently reported events were loss or illness of loved ones, divorce, and sexual abuse, with women reporting more often PTE/OLEs (particularly sexual abuse) than men. In the exposed population, the probable PTSD prevalence was 2.0%, with significantly higher odds among women, middle aged groups (45–54 years), people with a Moroccan or Turkish ethnicity, and subjects who reported “sexual abuse” as the most traumatic event. In addition, time was an important factor: when the event occurred less than four years ago, the odds of developing PTSD were about five times higher than when the event occurred more than nine years ago.

### 4.1. Gender

A recent exploratory study indicates that the twice higher PTSD prevalence in women as compared to men [35,36] exists consistently apart from cultural and socioeconomic country characteristics, suggesting that explanations for the higher risk of women are to be found elsewhere [37]. Many authors have shown that women are more likely than men to experience sexual abuse [36,38,39]. The present study confirms these assertions. Research repeatedly indicated that victims of intimately intrusive violence, like sexual abuse, develop PTSD at higher rates than those who are exposed to less intrusive violence [14,38,40,41]. However, when controlling for type of trauma experienced, differences by gender sustained. This finding is in line with other studies [36], for example, studies with men and women who experienced the same type of trauma, like disaster studies [42]. Consequently, other explanations must be explored to account for the differences between men and women, such as the emotional processing theory [43], which suggests that females create different trauma memory records, are more likely to blame themselves for the trauma, and view the world as more dangerous than male trauma victims (see also Van Zelst et al. [13]). In addition, women’s higher PTSD risk might also be due to other factors, like higher levels of peritraumatic dissociation, inadequate social support resources, or gender-specific acute psychobiological reactions to trauma [36]. In addition, a remarkable finding regarding gender was recently published: while women showed similar levels of PTSD symptoms after both PTEs (or A1 events) and OLEs (or non-A1 events), men reported even higher levels of PTSD symptoms after non-A1 than A1 events [44].

### 4.2. Ethnicity

Minority status has generally been associated with a greater risk for PTSD in civilians that are exposed to trauma, although the effect tends to vary considerably between studies [17,45]. The present study partially confirms this finding: the prevalence in people from Turkish or Moroccan descent was higher when compared to indigenous Dutch; however, the rate among other ethnic minority groups did not substantially deviate from the majority group.

Two of the largest non-EU immigrant populations in European countries are the Turkish and Moroccan communities [46]. Large Turkish immigrant and ethnic groups reside in Germany, Austria, the Netherlands, Belgium, Switzerland, Sweden, UK, and Denmark, making up 7.5% of the total foreign-born EU population, whereas the Moroccan groups found their way mainly to Spain, Italy, France, Belgium, The Netherlands, Germany, and UK, and they represent 5.8% of the total immigrant population [46].

Both of the groups migrated to Europe mainly as guest workers after World War II, mostly from underdeveloped rural areas, with little to no education, selected based on age, health, and physical condition [47,48].

Turkish and Moroccan immigrants display the lowest educational attainment and income levels, a high rate of unemployment (highest among first-generation women), and they occupy the most unstable jobs, sometimes in undocumented status immigrants from Morocco in particular [48]. They are also overrepresented in socially deprived neighborhoods, where access to high-quality education and health care is more challenging [49].

According to the ‘differential exposure and vulnerability hypotheses [50]’, ethnic minorities may be more exposed to potentially traumatic stressors and other life events, and more negatively impacted by trauma exposure when compared to majority groups [51,52]. Reviews on ethnic variation in PTSD prevalence have identified various sociocultural factors that may serve as potential mediators in the relationship between ethnicity and PTSD. These include ethnic variation in the tendency to experience a peritraumatic response, social disadvantage, adherence to cultural values, acculturative stress, differential expressive styles, and post trauma-coping [53]. In addition, Alegría et al. suggest that the interpretation of traumatic experiences might be quite diverse in different ethnic groups [54]. The combination with socioeconomic position and historical marginalization might play a role in the explanation of minorities’ elevated risk [55]. Nonetheless, the specific vulnerability of Moroccan and Turkish groups demonstrates that there are still some questions regarding the differences in PTSD prevalence in the ethnic minority groups.

### 4.3. Comparisons with Other Studies

It is difficult to detail comparisons between our data and data from previous studies because of the use of different methodologies, different diagnostic criteria, and the fact that most of the studies report lifetime or 12-month PTSD prevalence. Notwithstanding this limitation, there are a few findings that warrant attention. Table 4 shows the PTSD and PTE prevalence reported in three general population studies (based on the Composite International Diagnostic Interview, CIDI) and in three TSQ-based studies in populations that are affected by disaster. The prevalence of PTE/OLEs in our sample is somewhere between the results of two earlier Dutch CIDI-based population samples among adults [8,56], and in line with the PTE rate in, for instance, the United States. Table 4 includes the PTSD and PTE prevalence of the United States, not only because of the availability of TSQ-based disaster findings [28], but also because The Netherlands and the United States—like Australia, Canada, and New Zealand—were suggested to belong to a similar group in terms of socio-economic country vulnerability in combination with their PTE level, which is accompanied by a fairly high PTSD prevalence [2]). The PTE/OLE exposure, as presented in the current study, fits within this pattern.

Our reported TSQ-based PTSD prevalence is the same as the 12-month prevalence that was estimated by De Graaf and colleagues (2%), which is half of the lifetime prevalence reported in the same study (4.0%) [56]. This ratio matches the “lifetime is twice 12-month” rule of thumb that was suggested by Forbes and colleagues [57]), but it is more than three times lower than the 7.4% estimated by De Vries and Olff [8]. Another difference when compared to the study by De Vries and Olff is the notable difference in disaster exposure. The older study found a relatively high exposure of 8.4% and 4.0% to natural or man-made disasters in men and women, respectively. The disaster exposure in the present study is 0.6% (0.7% in men and 0.6% in woman), which fits within the bandwidth of disaster-related PTSD prevalence (0.0–3.8%) described in a recent cross-national comparison study [58].

All three of the TSQ-based studies show a dramatically higher prevalence in the disaster affected populations that remain to be high in the first year and seem to decrease after 1.5 to 2 years regardless of the differences in prevalence between the CIDI-based studies in Table 4 as suggested by Bonanno and colleagues [16]. The TSQ prevalence in the Dutch general population and the CIDI prevalence in the Netherlands and United States are somewhat similar. Based on this similarity, we carefully conclude that our TSQ prevalence can serve as a baseline for interpreting disaster-exposed subsamples with known risk factors, in a country like the Netherlands. The discussed disaster samples have limited data points, yet they initially show a higher prevalence that eventually decreases (as suggested by Bonanno et al. [16]). The higher part can only be partly explained by an earlier finding that self-report questionnaires are more prone to overestimation since the prevalence follows this pattern, which matches the idea of a gradual recovery.

### 4.4. Strengths and Limitations

This study is one of the few studies that examined PTSD symptomatology regarding a narrow time frame (one-week), which might be more accurate than a lifetime or 12-month prevalence, as the human memory is fallible and might be unreliable [59]. This memory bias might be higher in the assessment of lifetime exposure as compared to one-week symptoms. Besides the issue concerning the time since exposure, the issue of memory-bias in symptoms is smaller in 12-month estimates. Working with one-week prevalence is probably the most reliable approach of the three. The differences between time-periods in assessments and their consequences deserve further study.

In addition, the present findings were based on a large sample size that allows for stable estimates of the prevalence of PTSD symptoms. The additional value of the current study as compared with previous studies (e.g., the ESEMeD project) is in the high response percentage (65% [29]). Thus, the representation is satisfying, though some specific groups were somewhat underrepresented, like lower secondary educated people and subjects of Turkish or Moroccan origin. Moreover, we do not know whether cases with PTSD more often did not want to engage in the study.

Still, a number of caveats need to be acknowledged. First, in cross-sectional analyses, such as the present ones, inferences cannot be made about the directional relationship between the predictor and outcome. Furthermore, from the clinical point of view, although we used a validated and standardized symptom scale to assess PTSD, the results do not substitute for clinician-diagnosed psychopathology—in that sense, the TSQ is a rather limited instrument. In addition, all the data were based on recall. However lifetime prevalence is usually more biased than the one-week assessment used in the current study (see also De Graaf et al. [60]) and the 30-days assessment used by Kessler and colleagues after hurricane Katrina, as mentioned above [26].

Moreover, the participants met DSM-IV criterion A (which concerns the kind of exposure to the event and the nature of the response (horror)), to only a certain extent. A major proportion of PTE/OLEs that people indicated in this study as their worst event did not fulfill the DSM-IV A1 stressor criterion because life threatening was not always the case. We therefore measured both potentially traumatic events (PTEs) and other stressful life events (OLEs). We categorized loss following death under OLE, as these events are not strictly traumatic according to DSM. Only after a death caused by violence or abuse may loss be considered as traumatic. Furthermore, with regard to grief DSM-5 distinguished a different disorder in the rubric of trauma and stress related disorders: Persistent complex bereavement disorder. Loss after death of child and loss after death of partner both fall therefore in the OLE category. Unfortunately, most of the epidemiological studies included prevalence rates of loss events among the prevalence of traumatic experiences [1,61].

The revision of Criterion A1 in DSM-5 narrowed qualifying traumatic events, such that the unexpected death of family or a close friend due to natural causes is no longer included. In addition to the previously cited Van den Berg study [44], our data add to the discussion on the broadening of Criterion A1, especially to the question of the inclusion of loss of loved ones. The loss of loved ones does not fulfill the DSM-IV A1 Criterion or the DSM-5 A Criterion, although participants who were exposed to this event scored high on PTSD symptoms. A study that compared the prevalence rates of DSM-IV with DSM-5 showed that the DSM-5 prevalence estimates were slightly lower than their DSM-IV counterparts, although only two of these differences were statistically significant. The major reasons individuals met DSM-IV criteria, but not DSM-5 criteria, were the exclusion of non-accidental, non-violent deaths from Criterion A, and the new requirement of at least one active avoidance symptom [62,63]. Therefore, we recommend using TSQ in further research and clinical practice, always accompanied by a reference to the version adopted to enable comparisons, although a revision of the TSQ according to the DSM-5 criteria is advised.

### 4.5. Future Studies

This study is one of the first general population studies based on the TSQ. It is recommended to perform similar studies in representative samples in other countries, on the one hand to allow for cross-national comparisons, on the other to create baseline information for the interpretation of the screening results in particular samples that are affected by disasters, terrorism, or other impactful events. Additionally, future longitudinal studies should identify and verify risk and protective factors and use comprehensive assessments in the measurement of exposure to PTE/OLEs and PTSD. They should preferably adopt theory-driven models to formally test for interactions between sociocultural factors and PTSD-probability. In addition, other contributing factors to PTSD could be studied, such as social support and family background.

Moreover, a paucity of research exists regarding the effects of traumatic and other life events on people from non-Western culture. Finally, only a few of these predictors are found to be significantly associated to post-traumatic stress symptoms in the present study, despite the fact that many predictors had been suggested in prior literature as risk and protective factors in PTSD symptoms or diagnoses. These findings suggest that our understanding of vulnerability to post-traumatic stress symptoms is at an early stage and this leads us to the direction that factors unique to the combination of the person and the nature of the exposure are the determining factors in understanding who becomes symptomatic and who does not.

### 4.6. Practical Implications

As said before, the present study generated baseline information on the Dutch general population based on an alternative instrument than that applied in earlier studies. The prevalence estimated might have practical use in the assessment of the health impact of disasters and other calamities. From a more general public health promotion perspective, the results of the present study should be seen as encouragement for giving specific attention to women, ethnic minorities, and survivors of sexual abuse. There is striking heterogeneity in adaptation and adjustment after potentially traumatic and other life events, and this study also shows that the majority of affected people go on with their lives without experiencing substantial consequences [64,65]. Besides, our results showed that time was an important factor; it may be that ‘time can heal some wounds’ or people sought help earlier and their problems were solved. There is a need for greater appreciation of the role that is played by time elapsed since the exposure to trauma or other life events. A better understanding of the trajectories of reactions over time after exposure to PTE/OLEs can benefit public health planning and treatment [66].

## 5. Conclusions

Exposure to potentially traumatic and other life events is common (71.1%) in the Dutch-speaking general population of the Netherlands. The one-week prevalence of PTSD in this sample was 2.0 percent. The sociodemographic profile that was found to be strongly associated with PTSD is characterized by female gender, a background of Turkish or Moroccan ethnicity, and exposure to sexual abuse. In terms of public health care, prevention interventions should target these groups as priorities. The study provides potential reference material for future disaster mental health research.

## Figures and Tables

**Table 1:** Prevalence estimates of lifetime exposure to potentially traumatic and other life events in the Dutch population based on a sample of N = 6457.

Event	Male		Female		Total		Female vs. Male
	%	SE	%	SE	%	SE	OR (95% CI)
Potentially traumatic events							
Serious traffic accident	5.5	0.6	3.4	0.4	4.4	0.3	**0.60 (0.42–0.87)**
Illness of loved one *	6.1	0.6	8.2	0.6	7.1	0.4	**1.38 (1.07–1.78)**
Accident of loved one *	1.6	0.3	1.3	0.3	1.5	0.2	0.84 (0.44–1.61)
Violent assault	2.1	0.4	2.8	0.4	2.4	0.3	1.32 (0.84–2.08)
Physical abuse	1.9	0.4	1.9	0.2	1.9	0.2	0.98 (0.59–1.63)
Sexual abuse	0.9	0.2	4.0	0.3	2.5	0.2	**4.57 (2.69–7.78)**
Disaster	0.7	0.2	0.6	0.1	0.6	0.1	0.88 (0.48–1.60)
Exposure to war	1.0	0.2	0.9	0.2	1.0	0.1	0.93 (0.47–1.87)
Loss events							
Loss of child	2.5	0.4	2.4	0.3	2.4	0.2	0.99 (0.66–1.48)
Loss of partner	1.8	0.3	2.9	0.3	2.4	0.2	**1.59 (1.07–2.35)**
Loss of loved one (other than child or partner) *	25.2	1.0	27.6	1.2	26.4	0.8	1.13 (0.97–1.32)
Other life events							
Serious problems with loved one *	1.8	0.3	1.6	0.2	1.7	0.2	0.87 (0.52–1.46)
Not having children *	0.4	0.1	0.9	0.2	0.7	0.1	**2.14 (1.06–4.33)**
Serious relational problems *	2.2	0.3	2.7	0.4	2.4	0.3	1.25 (0.78–2.00)
Serious socio-economic problems *	1.1	0.3	0.5	0.1	0.8	0.1	**0.46 (0.24–0.89)**
Other	2.4	0.4	1.4	0.2	1.9	0.2	**0.60 (0.37–0.98)**
Total exposure							
Any exposure	68.3	1.4	74.0	1.0	71.1	0.9	**1.32 (1.14–1.53)**
No exposure	31.7	1.4	26.0	1.0	28.9	0.9	**0.76 (0.65–0.88)**

Note. Numbers in bold indicate significant gender differences on event, based on odds ratio and 95% confidence interval. * Other events and risks than Trauma Screening Questionnaire (TSQ) events as reported by respondents.

**Table 2:** The association between type of event and posttraumatic stress disorder (PTSD) (N = 4639).

	Exposed	PTSD	At Risk for PTSD Given Exposure
	%	%	OR (95% CI)
Potential traumatic events			
Serious traffic accident	6.3	0.1	0.52 (0.20–1.36)
Illness of loved one *	10.0	0.2	1.18 (0.58–2.40)
Accident of loved one *	2.0	0.0	0.77 (0.19–3.17)
Violent assault	3.4	0.2	2.45 (0.56–10.82)
Physical abuse	2.7	0.0	0.43 (0.07–2.76)
Sexual abuse	3.4	0.2	**3.52 (1.64–7.52)**
Disaster	0.9	0.0	--
Exposure to war	1.3	0.1	2.02 (0.63–6.47)
Loss events			
Loss of child	3.5	0.1	0.90 (0.28–2.85)
Loss of partner	3.3	0.1	1.40 (0.68–2.87)
Loss of other loved one *	37.2	0.4	**0.44 (0.21–0.91)**
Other life events			
Divorce	12.0	0.1	**0.46 (0.22–0.97)**
Dismissal	3.4	0.1	2.00 (0.71–5.65)
Serious problems with loved one *	2.4	0.1	1.10 (0.38–3.17)
Not having children *	1.0	0.0	--
Serious relational problems *	3.4	0.3	**4.75 (1.83–12.34)**
Serious socio-economic problems *	1.2	0.0	1.97 (0.52–7.51)
Other *	2.7	0.0	0.71 (0.21–2.43)

Note. Weighted percentages, odds ratios and 95% confidence intervals for being at risk for PTSD given exposure (six or more TSQ items endorsed); Numbers in bold indicate significant odds ratios. -- Odds ratios could not be estimated due to an absence of PTSD cases. * Other events and risks than TSQ events as reported by respondents.

**Table 3:** The association between sociodemographic characteristics, event type and being at risk for PTSD with and without correction for time since event (adjusted odds ratios and 95% confidence intervals from logistic regression analysis with all variables in the model, N = 4545).

				Time Since Event
	%	% at Risk	AOR (95% CI)	AOR (95% CI)
Gender				
Female	51.3	2.2	**2.67 (1.50–4.76)**	**2.65 (1.50–4.67)**
Male	48.7	1.0	1	1
Age in years				
18–24	11.0	4.5	**3.36 (1.27–8.92)**	1.90 (0.68–5.29)
25–34	18.2	1.4	1.34 (0.56–3.24)	0.94 (0.40–2.20)
35–44	24.6	2.0	**2.13 (1.03–4.38)**	1.72 (0.85–3.47)
45–54	25.3	2.2	**2.48 (1.16–5.28)**	**2.28 (1.07–4.84)**
55–64	20.8	1.2	1	1
Education				
Primary	7.0	5.2	1.61 (0.68–3.85)	1.68 (0.72–3.91)
Lower secondary	22.1	3.1	1.07 (0.53–2.16)	1.06 (0.52–2.14)
Higher secondary	42.0	1.2	0.50 (0.25–1.00)	**0.46 (0.23–0.93)**
Higher professional	28.9	1.8	1	1
Paid job				
Yes	77.2	1.5	1	1
No	22.8	3.9	1.91 (0.99–3.68)	**1.97 (1.05−3.68)**
Ethnicity				
Dutch	85.2	1.7	1	1
Moroccan	0.5	15.9	**6.56 (1.52–28.23)**	**6.97 (1.21–40.27)**
Turkish	0.7	18.9	**10.78 (2.61–44.53)**	**12.41 (3.16–48.72)**
Surinam	1.8	1.2	0.32 (0.44–2.34)	0.31 (0.04–2.54)
Antillean	0.7	6.7	3.74 (0.57–24.42)	3.52 (0.48–26.11)
Indonesian	3.3	1.9	0.82 (0.20–3.28)	0.95 (0.23–3.92)
Other western	5.4	1.5	0.95 (0.39–2.32)	1.02 (0.41–2.49)
Other non-western	2.2	6.3	2.60 (0.47–14.57)	2.50 (0.47–13.40)
Potentially traumatic events				
Serious traffic accident	6.4	0.1	0.35 (0.05–2.45)	0.48 (0.07–3.04)
Illness of loved one	10.2	0.2	1.18 (0.30–4.63)	0.93 (0.24–3.64)
Accident of loved one	2.1	0.0	0.77 (0.11–5.16)	0.78 (0.12–5.30)
Violent assault	3.5	0.2	1.75 (0.38–8.17)	1.75 (0.39–7.91)
Physical abuse	2.7	0.0	0.33 (0.04–2.90)	0.37 (0.05–2.67)
Sexual abuse	3.5	0.2	2.54 (0.62–10.30)	**5.34 (1.31–21.70)**
Exposure to war	1.3	0.1	0.68 (0.06–8.33)	0.89 (0.06–12.65)
Loss events				
Loss of child	3.5	0.1	0.94 (0.19–4.62)	1.26 (0.26–6.06)
Loss of partner	3.3	0.1	1.54 (0.38–6.25)	1.32 (0.32–5.44)
Loss of other loved one	37.9	0.4	0.57 (0.15–2.18)	0.55 (0.14 −2.12)
Other life events				
Divorce	12.2	0.1	0.53 (0.14–2.03)	0.68 (0.18–2.63)
Dismissal	3.5	0.1	2.13 (0.48–9.50)	1.62 (0.37–7.09)
Serious problems with loved one	2.4	0.1	1.08 (0.23–5.10)	1.11 (0.24–5.18)
Serious relational problems	3.5	0.3	3.26 (0.83–12.82)	3.24 (0.83–12.64)
Serious socio-economic problems	1.2	0.0	2.85 (0.41–19.90)	2.36 (0.33–17.14)
Other	2.7	0.0	1	1
Years passed since event				
<4 years	31.5	1.0	-	**4.83 (2.39–9.78)**
4–9 years	22.0	1.6	-	1.57 (0.60–4.13)
>9 years	46.6	1.0	-	1

Note. Weighted percentages, odds ratios and 95% confidence intervals for being at risk for PTSD given exposure (6 or more TSQ items endorsed); Numbers in bold indicate significant odds ratios.

**Table 4:** Several Composite International Diagnostic Interview (CIDI)-based general population studies and TSQ-based disaster studies: PTSD prevalence.

	Sample (N)	Age	PTSD Prevalence (%)
CIDI-based general population studies			
United States [7]	5692	18+	Lifetime: 6.8 (3.6 in men, 9.7 in women)
Netherlands-1 [56]	1094	18+	Lifetime: 4.0; 12-month: 2.5
Netherlands-2 [8]	1087	18–80	Lifetime: 7.4 (4.3 in men, 8.8 in women)
TSQ-based disaster studies			
London Bombings [25]	596	NA	1-week: 50.7 (median nearly 7 months after bombings), 29.1 (on average 10–11 months), 17.6 (on average 17 months), 9.1 (on average 22 months)
Hurricane Katrina [26]	815	18+	New Orleans Metropolitan, 30-days: 25.9 (5–8 months after hurricane), 24.1 (1 year later) Remainder of sample, 30-days: 11.8 (5–8 months after hurricane), 20.0 (1 year later)
Plane crash Schiphol [27]	121	18+	1-week: 46 (2 months after plane crash), 47 (9 months after plane crash)

Note. N = Number of respondents, NA = Not available.
